# Mechanisms of processing speed training and transfer effects across the adult lifespan: protocol of a multi-site cognitive training study

**DOI:** 10.1186/s40359-022-00877-7

**Published:** 2022-07-08

**Authors:** Claudia C. von Bastian, Alice Reinhartz, Robert C. Udale, Stéphanie Grégoire, Mehdi Essounni, Sylvie Belleville, Tilo Strobach

**Affiliations:** 1 Department of Psychology, University of Sheffield, 1 Vicar Lane, Sheffield, S1 2LT UK; 2 Medical School Hamburg, Hamburg, Germany; 3 Centre de Recherche de L’Institut Universitaire de Gériatrie de Montréal (CRIUGM), Montréal, Canada; 4 Université de Montréal (UdeM), Montréal, Canada

**Keywords:** Study protocol, Cognitive training, Processing speed, Attentional control, Diffusion modeling, Drift rate, Latent-variable modeling, Adult Lifespan, Training gains, Transfer gains

## Abstract

**Background:**

In recent years, cognitive training has gained popularity as a cost-effective and accessible intervention aiming at compensating for or even counteracting age-related cognitive declines during adulthood. Whereas the evidence for the effectiveness of cognitive training in general is inconsistent, processing speed training has been a notable successful exception, showing promising generalized benefits in untrained tasks and everyday cognitive functioning. The goal of this study is to investigate why and when processing speed training can lead to transfer across the adult lifespan. Specifically, we will test (1) whether training-induced changes in the rate of evidence accumulation underpin transfer to cognitive performance in untrained contexts, and (2) whether these transfer effects increase with stronger attentional control demands of the training tasks.

**Methods:**

We will employ a multi-site, longitudinal, double-blinded and actively controlled study design with a target sample size of *N* = 400 adult participants between 18 and 85 years old. Participants will be randomly assigned to one of three processing speed training interventions with varying attentional control demands (choice reaction time, switching, or dual tasks) which will be compared to an active control group training simple reaction time tasks with minimal attentional control demands. All groups will complete 10 home-based training sessions comprising three tasks. Training gains, near transfer to the untrained tasks of the other groups, and far transfer to working memory, inhibitory control, reasoning, and everyday cognitive functioning will be assessed in the laboratory directly before, immediately after, and three months after training (i.e., pretest, posttest, and follow-up, respectively). We will estimate the rate of evidence accumulation (drift rate) with diffusion modeling and conduct latent-change score modeling for hypothesis testing.

**Discussion:**

This study will contribute to identifying the cognitive processes that change when training speeded tasks with varying attentional control demands across the adult lifespan. A better understanding of how processing speed training affects specific cognitive mechanisms will enable researchers to maximize the effectiveness of cognitive training in producing broad transfer to psychologically meaningful everyday life outcomes.

*Trial registration* Open Science Framework Registries, registration https://doi.org/10.17605/OSF.IO/J5G7E; date of registration: 9 May 2022.

## Background

Fluid cognitive abilities such as processing speed, attentional control, and reasoning decline with progressing age [1]. In later adulthood, these profound changes can affect everyday life functioning and life satisfaction [2]. Thus, it is critical to identify evidence-based and accessible interventions that can counteract these declines. In recent years, cognitive training interventions have gained popularity as a cost-effective and easy-to-administer option for maintaining cognitive health into later life. The rationale of cognitive training is straightforward: By targeting and improving specific core cognitive processes, improvements in these basic cognitive processes can generalize—or transfer—to untrained contexts also drawing on these processes. However, the empirical evidence for the effectiveness of cognitive training in eliciting such broad transfer effects is overall weak [3]: Although most training interventions induce large and robust performance gains in the trained tasks [4], these training effects often do not transfer to untrained tasks or everyday life functioning [5, 6]. A notable successful exception appears to be training interventions targeting processing speed and attentional control, which have been shown to transfer to older adults' everyday functioning and may even delay the onset of dementia [for a review, see 7]. These successful training interventions typically require participants to react as quickly and as accurately as possible to a stimulus, often in combination with attentional control demands such as the inclusion of a secondary task. However, the cognitive mechanisms underlying these training and transfer effects are yet unclear [3, 8]. The goal of this study is to identify the key mechanisms of when and why processing speed training can elicit transfer.

### Attention control demands in processing speed tasks

The speed with which people process information is a key cognitive ability that is strongly related to a wide range of cognitive domains [9], including working memory [10] and fluid intelligence [11]. Moreover, declines in processing speed have been proposed to account for much of the cognitive changes observed in aging [12]. Hence, interventions successful in increasing speed have great potential to yield broad cognitive benefits. Yet, tentative evidence suggests that targeting processing speed alone may not suffice for inducing transfer effects [13, 14]. Indeed, successful speed training interventions [7, 15] often additionally demand attentional control, which is the ability to maintain an operative goal, and goal-relevant information, in the face of distraction [16].

Existing processing speed training tasks vary in the extent to which they involve attentional control. For example, a typical processing speed task with minimal attentional control demands is the simple reaction time (RT) task, in which participants are asked to press a key as soon as a stimulus appears on screen. In contrast, in a choice RT task, participants are asked to decide between two alternatives, such as whether a simple geometric shape is blue or green, as quickly and as accurately as possible. Different to simple RT tasks, choice RT tasks require goal maintenance and distraction avoidance [17], although these attentional control demands are still relatively low as participants can focus their attention on a single task.

Attentional control demands are higher when multitasking [18], that is, when two (or more) tasks are processed sequentially (switching tasks) or simultaneously (dual tasks). In task switching, multiple choice RT tasks are performed sequentially, often in random order with a cue indicating the upcoming task (task-cueing paradigm). For example, participants may be asked to switch between categorizing simple geometric shapes by their color (e.g., deciding whether the object is blue or green) or by their shape (e.g., deciding whether the object is curvy or spiky). Switching between the two tasks requires focusing on the currently relevant task while inhibiting the other, currently no longer relevant task, a process which involves maintaining, updating, inhibiting, and activating a different task in working memory [19, 20] as well as using proactive control strategies [21]. In dual tasks, two tasks are presented and performed simultaneously; for example, participants are instructed to make both decisions related to color and shape on the same stimulus at the same time. Hence, in addition to the attentional demands of task switching, participants need to divide their attention between the two tasks, which requires task coordination and optimizing attentional resource allocation between two simultaneous and independent task processing streams and their concurrence for limited processing capacities [15, 22]. Yet, we do not know conclusively how the attentional control demands of a task affect the effectiveness of training in producing broad transfer gains. Therefore, in this study, we will systematically compare the effects of training with choice RT tasks, switching tasks, and dual tasks to an active control group practicing simple RT tasks. If attentional control demands are critical in inducing broad transfer, we would expect to find larger transfer gains the greater the training tasks’ attentional control demands.

### Estimating cognitive processes underpinning processing speed performance: the diffusion model

Processing speed performance is commonly measured with mean RTs and/or the proportion of correct responses (accuracy) in speeded tasks. However, relying only on mean RTs and accuracy when evaluating processing speed gains during training is problematic for at least three reasons. One concern is the psychometric validity of these measures. Specifically, RT distributions typically have a positively skewed, asymmetric shape. Therefore, the mean and other point estimates are a poor representation of that distribution. Accuracies are not a good alternative either, because adults tend to perform near ceiling in these tasks and, thus, usually only little variance in performance is observed. A second concern is that people may differ in their speed-accuracy trade-off, that is, the extent to which they emphasize giving speedy or accurate responses. Furthermore, these preferences may change across the lifespan and over time in a training context. This complex relation between the speed and accuracy distribution complicates their interpretation. Finally, mean RTs and accuracies do not directly assess the psychological processes assumed to underlie the decision-making process.

**Fig. 1:**
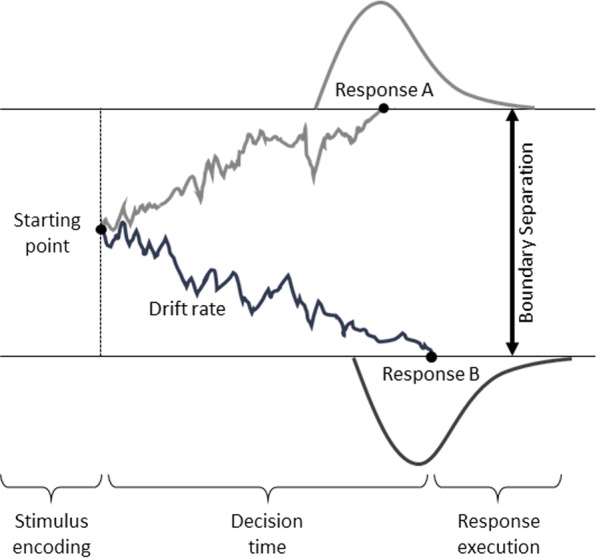
Simplified Illustration of the Diffusion Model and Its Main Parameters. *Note*. After stimulus encoding, information accumulation begins at the starting point *z* and proceeds with a mean drift rate *v* until either response option A or B is reached, followed by response execution. The observed RT is the sum of the decision time and the time required for non-decision processes (*Ter*) such as stimulus encoding and response execution

The diffusion model (Fig. 1) is a computational approach that addresses these three concerns and estimates parameters representing the psychologically meaningful processes involved in speeded cognitive tasks [23]. This model assumes that each decision involves continuously accumulating information—a mix of evidence and noise—that is indicative of two alternative options. Once the stimulus has been encoded, the decision-making process begins at a starting point *z*, with the drift rate *v* reflecting the rate of accumulating evidence until a threshold is reached. The decision-making process ends when this threshold is surpassed and, thus, a critical upper or lower boundary is reached, resulting in executing the response. The boundary separation *a* reflects response caution: People who tend to respond more conservatively (i.e., base their decision on more evidence) will have wider boundaries, whereas the boundary separation will be narrower in people who tend to respond more liberally. To map task performance onto these processes, computational routines are used to estimate the parameters by fitting the model to a data set. Critically, the diffusion model jointly considers RTs and accuracy, thereby accounting for individual differences and change in speed-accuracy trade-offs.

Decomposing task performance into theoretically meaningful diffusion model parameter estimates can provide valuable insights into the mechanisms of transfer across the lifespan. First, the diffusion model parameters reflect trait-like properties of cognitive processes that are temporally stable and task-general [24], yet they are sensitive to age-related differences [25] and manipulations of attentional control demands [26]. These properties render the diffusion model parameters ideally suitable for investigating associations between intervention-specific effects and individual differences. Furthermore, previous research has shown that individual differences in drift rate are associated with mental abilities such as fluid intelligence [10, 27], making it a prime candidate mechanism potentially underlying the transfer effects observed in previous processing speed training studies. In the present study, we will be able to directly test this hypothesis by distinguishing training-induced changes in drift rate from such in response caution and non-decision components and evaluate how change in these cognitive processes contribute to transfer effects.

This preregistered study will follow methodological best practices [28–30] by including an active control group to account for test–retest and placebo effects, using multiple tasks to assess abilities on the latent level, and testing a sample large enough to provide adequate statistical power. Furthermore, our conclusions will be based on Bayesian inference, allowing for gauging the strength of evidence for the presence as well as the absence of any hypothesized effects and relationships.

### Objectives

The overarching goal of this study is to identify the cognitive processes improved by training speed tasks and how they are affected by varying attentional control demands across the adult lifespan. This study goes beyond previous cognitive training studies by using diffusion modeling to estimate theoretically and psychologically meaningful components of information processing and relating them to gains in untrained contexts. For this purpose, we pursue the following objectives:

1. Examine how extensive speed training affects the cognitive processes involved as reflected by changes in diffusion model parameters, and whether these changes in cognitive processes vary across the lifespan.
2. Investigate how changes observed in these cognitive processes are related to transfer effects in working memory, inhibitory control, reasoning, and everyday cognitive functioning, and whether these changes are maintained after the end of the training intervention.
3. Determine how attentional control demands of training interventions modulate these effects.

### Hypotheses

#### Hypothesis 1

(training gains): On average, participants in the three experimental conditions will show larger pre-post improvements in their respective training tasks than participants in the active control condition.

#### Hypothesis 2

(near transfer gains): On average, participants in the three experimental training conditions will show larger pre-post improvements in the untrained processing speed training tasks of the other experimental conditions than participants in the active control condition.

#### Hypothesis 3

(far transfer gains): On average, participants in the three experimental training conditions will show larger pre-post improvements in working memory, inhibitory control, reasoning, and everyday cognitive functioning than participants in the active control condition.

#### Hypothesis 4

(attentional control demands): Compared to the active control condition, experimental training conditions with stronger attentional control demands will induce larger near and far transfer effects. Specifically, transfer effects will be largest after dual task training, followed by switching training, and, lastly, choice RT training.

#### Hypothesis 5

(underlying mechanism): Training-induced changes in drift rate in the experimental training conditions relative to the active control condition predict the size of near and far transfer effects, and the relationship between training-induced change in drift rate and the size of transfer effects will be stronger with increased attentional control demands of the training condition. Specifically, we expect this relationship to be strongest after dual task training, followed by switching training, and, lastly, choice RT training.

## Method

This study protocol is preregistered on the Open Science Framework at https://osf.io/j5g7e.

### Design

Table 1 provides an overview of the study design. This multi-site, longitudinal, double-blinded, and actively controlled study uses a mixed 4 (training condition, between-subjects: simple RT, choice RT, task switching, and dual task training) × 3 (time, within-subjects: pretest, posttest, follow-up) factors design with age as covariate.

**Table 1:** Schedule of enrollment, assessments, and interventions

Measure	Study phase and timepoint									
	Pre-assessment	Pretest				Intervention	Posttest		Follow-Up	
	T0–P	T0–Q	T1–V1	T1–V2	T1–R	S1–S10	T2–V1	T2–V2	T3–V1	T3–V2
*Enrolment*										
Eligibility screening and conditional enrolment	X									
Informed consent^a^		X	X							
MoCA assessment, confirmation of eligibility, and final enrolment			X							
Randomization					X					
*Interventions*										
Active control: Simple RT						X				
Experimental—low: Choice RT						X				
Experimental—medium: Switching						X				
Experimental—high: Dual tasking						X				
*Assessment of Training and Near Transfer Gains*										
Simple RT tasks (drawings, shapes, numbers)				X				X		X
Choice RT tasks (drawings, shapes, numbers)				X				X		X
Switching tasks (drawings, shapes, numbers)				X				X		X
Dual tasks (drawings, shapes, numbers)				X				X		X
*Assessment of Far Transfer Gains*										
Working memory										
Updating			X				X		X	
Binding			X				X		X	
Continuous reproduction			X				X		X	
Inhibitory control										
Go/no-go			X				X		X	
Number stroop			X				X		X	
Simon			X				X		X	
Reasoning										
Matrix reasoning			X				X		X	
Paper folding			X				X		X	
Letter sets			X				X		X	
Everyday cognitive functioning										
Cognitive Failures Questionnaire (CFQ)			X				X		X	
Questionnaire d’Auto-évaluation de la Mémoire (QAM)			X				X		X	
Everyday Problems Test (EPT)			X				X		X	
*Assessment of Individual Characteristics*										
Socioeconomic background		X								
Cognitive training experience and motivation		X								
Computer and internet literacy		X								
Self-rated physical health		X								
General self-rated health			X				X		X	
Depression, Anxiety and Stress Scale—21 Items (DASS-21)		X								
Activities of Daily Living-Prevention Instrument (ADL-PI)			X				X		X	
Leisure activities		X								
Active driving		X								
Big Five Inventory (BFI)		X								
Short Grit Scale (Grit-S)		X								
Theories of Intelligence Scale (TIS)			X				X		X	
Exercise Self-Efficacy Scale (EXSE)		X								
General Self-Efficacy Scale (GSE)			X				X		X	
Perceived training benefits										X
Training review										X
Daily factors			X	X		X	X	X	X	X

*T* time, *P* phone screening, *Q* questionnaires, *V* visit to the laboratory, *R* randomization, *S* session, *MoCA* Montreal Cognitive Assessment, *RT* reaction time

^a^Participants in Sheffield will give consent online before completing the pre-assessment questionnaires, and participants in Hamburg and Montréal will give consent at their first visit to the laboratory

To test our hypotheses, we will compare three experimental training interventions with systematically increasing attentional control demands to an active control group practicing tasks involving only minimal attentional control demands. Participants ranging in age from young to old adulthood (18–85 years) will complete 10 sessions of either simple RT (active control), choice RT (training with low attentional control demands), switching (training with medium attentional control demands), or dual task training (training with high attentional control demands). We will assess near transfer to the respective other training tasks and far transfer to working memory, inhibitory control, reasoning, and everyday cognitive functioning before, immediately after, and three months after training (i.e., pretest, posttest, and follow-up, respectively). All training groups will undergo the same study procedures and be exposed to the same task materials, with the active control group serving as baseline for training-related changes in the cognitive abilities tested at pretest, posttest, and follow-up.

### Timeline

**Fig. 2:**
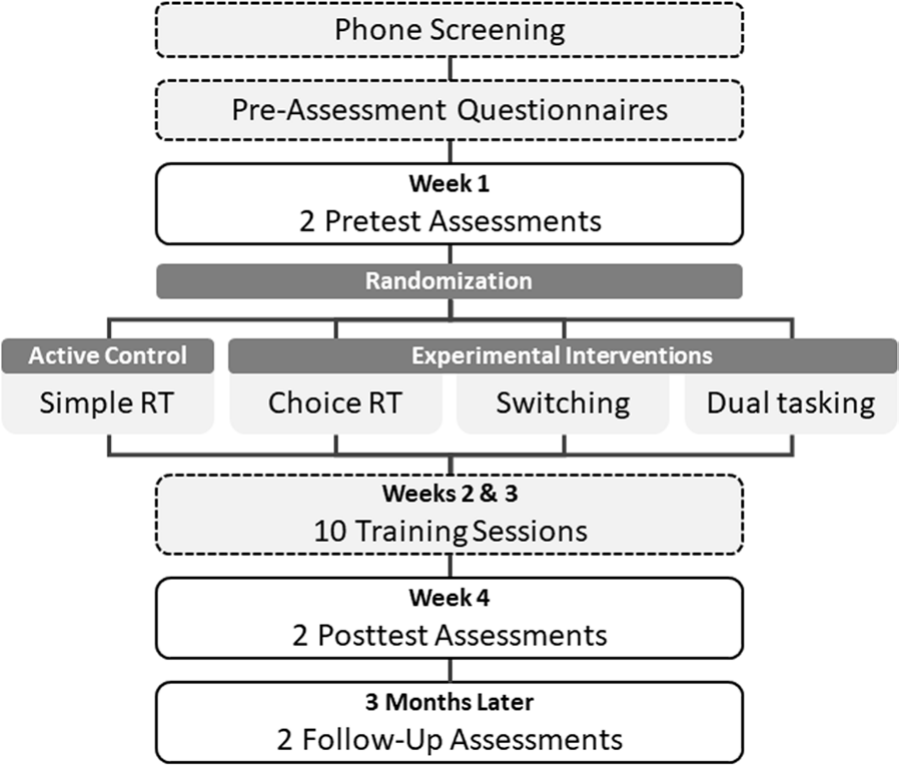
Study Timeline. *Note*. Dashed lines indicate elements of the study that participants (18- to 85-year old healthy adults) complete from home, and solid lines indicate study elements that participants complete in the laboratory at one of the three respective study sites (University of Sheffield, Medical School Hamburg, and Centre de Recherche de l’Institut Universitaire de Gériatrie de Montréal, CRIUGM)

Figure 2 provides an overview of the study’s timeline. Participation in this study will span approximately four months. After study sign-up and a phone screening confirming eligibility, participants will complete a set of short questionnaires from home at any time before their first visit to the laboratory (pre-assessment questionnaires). Each pretest, posttest and follow-up assessments consist of two test sessions (approximately 2 h each) that are completed within one week in the research laboratories of the participating institutions. After the second pretest session, participants will be asked to complete ten training sessions at home, each taking approximately 30 min, over the course of two weeks. Participants will complete the two posttest sessions in the following week and the two follow-up assessments three months later.

### Participants

Participants will be recruited from local communities for a study on “brain training” (German: “Übungen zum Gehirntraining”; French: “exercices pour stimuler le cerveau”) through a range of sources such as posters, leaflets, social media (e.g., Facebook, Twitter), outreach events (e.g., public lectures), ads in newspapers, and existing participant pools. Participants will be reimbursed after study completion (GBP 125 in Sheffield, EUR 150 in Hamburg, and CAD 250 in Montréal) or pro rata in case they withdraw consent and/or drop out of the study (GBP 12.50, EUR 15, or CAD 25, respectively for each visit, and GBP 50, EUR 60, or CAD 100 respectively, for completing the training phase). Psychology students at the University of Sheffield and the Medical School Hamburg can choose to receive a mix of course credits and monetary compensation. This study has been ethically approved by the institutional review boards at each of the three research sites. All participants will have provided written informed consent and have the right to withdraw from the study at any time until participation completion without any negative consequences.

#### Inclusion and exclusion criteria

Participants will self-report whether they meet the inclusion and exclusion criteria during a pre-scripted phone screening, except their score in the Montreal Cognitive Assessment [MoCA; 31]. The MoCA (version 8.1) will be administered as a pen-and-paper test at the beginning of the first pretest session.

Inclusion criteria:

- Age: 18–85 years old
- Access to a computer/laptop with internet connection
- Fluency in English (Sheffield), German (Hamburg), or French (Montréal), respectively
- Be in good health, that is, feeling physically fit to participate in the study and not currently diagnosed with any illness(es) that may limit their ability to participate in this study
- Commitment to take part in the entirety of the study, that is, willing to invest 1 month of active participation and a follow-up assessment 3 months later, with a total of 6 visits at the respective testing center

Exclusion criteria:

- MoCA score below 26^1^
- Color blindness
- Daily use of drugs (e.g., cannabis) and/or consumption of more than about one glass of alcohol (Sheffield: 25 units per week; Hamburg: 1 glass daily; Montréal: 1 unit daily)
- Knowledge of a current diagnosis with disease(s) that may limit participation in this study, including neurodegenerative diseases or dementia, neurological disorders, mental illness, other disease diagnoses known to the subjects which may impact the ability to take part in the study and/or knowledge of other brain diseases that may affect cognition and/or motor function of the hands
- Currently participating in another research project that could interfere with this project
- Previous participation in a research project on memory and/or cognitive training that could interfere with this project

#### Sample size

We aim for a total sample size of at least 400 participants (*n* = 100 per condition) recruited across the three sites. To account for stratification (see below), our goal is to recruit *n* = 136 participants per site (*N* = 408). Based on previous cognitive training studies, training effects tend to be very large, whereas transfer effects tend to be small. The required sample size to achieve 80% power at α = 0.05 for a small effect size (Cohen’s *f* = 0.10) is *N* = 280 for a main effect of condition on change in transfer measures from pretest to posttest. However, in this study, an additional aim is to determine how changes in cognitive processes during training (i.e., the diffusion model parameters) relate to changes in the transfer measures using multi-group latent-change score models, a structural-equation modeling technique. Currently, no empirical simulation studies exist offering guidelines for optimal sample sizes [32]. We therefore estimated the required sample size based on previous studies using similar techniques [33, 34] and general power considerations for correlational designs. According to simulation studies [35], correlations of *r* = 0.24, as are typically found for the relationship between processing speed and fluid intelligence [11], tend to stabilize at sample sizes between *N* = 304 to *N* = 341 (90% power, corridor of stability w = 0.10). The total sample size of *N* = 408 participants will allow for some attrition (~15 to 20%).

### Randomization

After the two pretest sessions and before the first training session, participants will be randomly assigned to one of the four conditions. The study will be conducted double blinded, that is, neither participants nor assessors will be aware of group affiliation.

Before the start of recruitment, a stratified list of group assignments is randomly generated centrally at the Sheffield site by an unblinded research team member, using the *sample()* function in R. To ensure an even distribution of the four groups, the list is generated in blocks of four group assignments at a time. Following this pre-generated list, participants within each stratum (age and gender) who completed the second pretest session will be randomly allocated to one of the four groups for each study site separately to maximize equal distribution of these characteristics in each training condition and study site. For stratification purposes only, we will form age groups that are about equally distributed across age decades:

- Ages 18–29 years: 24 participants
- Ages 30–39, 40–49, 50–59, and 60–69 years: 20 participants each
- Ages 70–85 years: 32 participants

Participants who completed both pretest sessions and were allocated a training condition but have not yet started their training intervention, as evidenced by not having opened the training tasks, will be replaced by new participants until the desired sample size is reached.

### Procedure

Data collection will take place at three sites: The University of Sheffield (United Kingdom), the Medical School Hamburg (Germany), and the Centre de Recherche de l'Institut Universitaire de Gériatrie de Montréal (CRIUGM, Canada). Detailed study coordinator, phone screening, and experimenter handbooks with equivalent versions in each language will ensure similar testing conditions and procedures across the three study sites.

All instructions, questionnaires, and stimulus materials were first set up in English, partially based on existing task instructions used in previous studies [5, 6, 36]. Once agreed upon, English instructions were then carefully translated into French and German by native speakers. All translations were double-checked by at least one other native speaking researcher of the respective language. Where available, we use published translations of validated instruments. All materials, in all language versions, were pilot tested at each site before data collection.

Participants will complete the 10 sessions of computer-based training self-administered at home with Tatool Web [www.tatool-web.com, 37]. Tatool Web is an open-source, freely available software based on JavaScript, HTML5 and CSS which runs through a web browser without requiring participants to install any additional software. Each session begins with a short questionnaire about daily factors that may impact cognitive performance, followed by three training tasks using different stimuli sets. After each training session, training data will be automatically uploaded to a web server, allowing for constant monitoring of participant commitment. We used this self-administered training procedure extensively and successfully in our previous research [5, 38, 39] including in studies with older adults [4, 6]. This home-based procedure has a range of benefits. It does not only save financial and time resources that would otherwise be needed for conducting 10 training sessions in the laboratory, the more realistic setting in the home environment also increases the ecological validity of the training regimen [40]. To counter the potential loss of experimental control, we will alert participants that their training data are constantly monitored and apply procedures to detect obvious irregularities in session durations and accuracy in task performance. Moreover, we will be in regular contact with the participants, and participants can always ask for support in case of technical difficulties. Furthermore, after training, we will ask participants to confirm that they have completed training in an undisturbed environment on a computer or laptop.

At pretest, posttest and follow-up, participants will be tested individually in two assessment sessions each in the laboratory. An experimenter will be available for questions and support during the assessment sessions. Table 2 lists the order in which we administer the measures to all participants at the two pretest, posttest, and follow-up sessions. Performance-based tasks and the Daily Factors Questionnaire will be administered with Tatool Web, and all other questionnaires will be administered via LimeSurvey (www.limesurvey.org). LimeSurvey is an open-source, freely available web application running through standard web browsers.

**Table 2:** Test order used at each assessment session

Assessment	
Session 1	Session 2
MoCA (pretest only)	Daily Factors Questionnaire
Questionnaires	Drawings
General self-rated health	Simple RT
General self-efficacy (GSE)	Choice RT (animacy, size)
Theories of intelligence (TIS)	Switching
Activities of daily living-prevention instrument (ADL-PI)	Choice RT (size, animacy)
Cognitive Failures Questionnaire (CFQ)	Dual task
Questionnaire d’Auto-évaluation de la Mémoire (QAM)	Shapes
Daily Factors Questionnaire	Simple RT
Everyday problems test	Choice RT (color, shape)
Matrix reasoning	Switching
Stroop	Choice RT (shape, color)
Continuous reproduction	Dual task
Paper folding	Numbers
Simon	Simple RT
Binding	Choice RT (parity, magnitude)
Letter sets	Switching
Go/no-go	Choice RT (magnitude, parity)
Updating	Dual task
	Training review (posttest and follow-up only)

Each assessment session takes approximately 2 h. Both assessment sessions will be administered at pretest, posttest, and follow-up

*RT* reaction time

### Training interventions

The four groups will practice tasks with three different stimuli sets (drawings, shapes, and numbers). Presenting identical stimuli across all four conditions has the benefit that potential perceptual or motivational effects of materials are held constant across groups. Furthermore, these stimuli sets have been used extensively in previous research and have shown excellent psychometric properties including high reliabilities in single-task and multiple-task situations [4–6, 19, 36, 39]. The *drawings* stimuli set comprises 20 line-drawings [41, 42] that are either animals (e.g., a giraffe) or objects (e.g., a comb), and either smaller (e.g., a comb) or larger than a soccer ball (e.g., a giraffe). The *shapes* stimuli set contains 20 simple geometric shapes that are either blue or green and either curvy or spiky. In the *numbers* set, stimuli are one-digit numbers (1–9 excluding 5) which are either odd or even and smaller or greater than five. As all stimuli are bivalent, they afford two task sets each (e.g., the drawings stimuli set affords an animacy and a size task). Therefore, these stimuli sets can be used to assess performance in both single-task and multiple-task situations. In each session, participants complete the three training tasks using these stimuli sets always in the same order (drawings first, then shapes, then numbers).

For each set of stimuli, participants will complete four blocks of 80 trials each (320 trials in total per task per session). After each trial, participants will receive feedback on the accuracy of their response, which is displayed for 250 ms. If no response is given within 10 s, the response is recorded as an omission error, and the next trial is presented. Each block will be preceded by a brief instruction to remind participants of the task and stimulus–response bindings, and a visual countdown. Participants can take short rests between blocks and tasks. Dependent measures of all training tasks will be accuracy and RT, which will be used to estimate the diffusion model parameters.

#### Simple RT tasks (active control condition)

Participants are asked to react as soon as a stimulus appears on the screen by pressing the space key on a computer keyboard. Stimulus appearance is preceded by a random jitter, ranging from 150 to 1170 ms. Within each block, stimuli are randomized, with each stimulus appearing equally often.

#### Choice RT tasks (low attentional control condition)

Participants are asked to classify the centrally presented stimulus as quickly and as accurately as possible by pressing the A or L key. For each trial, a visual cue is presented for 150 ms before stimulus onset until a response is given to remind participants of the task set (e.g., animacy for the decision whether a drawing shows an animal or an object). For each stimuli set, the two task sets are presented sequentially (AABB). Specifically, for the *drawings* set, participants will first complete two blocks of the animacy task set (“does the drawing show an animal or object?”), followed by two blocks of the size task set (“does the drawing show something smaller or larger than a football?”). For the *shapes* set, two blocks of the color task set (“is the shape blue or green?”) are followed by two blocks of the shape task (“is the shape curvy or spiky?”). Finally, for the *numbers* set, two blocks of the parity task (“is the number divisible by 2 or not?”) are followed by two blocks of the magnitude task (“is the number smaller or greater than 5?”). Within each block, the sequence of trials is randomized, with equal distributions of each stimulus and correct response.

#### Switching tasks (medium attentional control condition)

Participants are asked to perform two choice RT task sets (e.g., switching between classifying drawings based on their animacy or their size) in a random order, thereby requiring participants to flexibly adjust to changing environmental demands. Using a task-cueing paradigm, the currently relevant task set is indicated by a visual cue that is presented for 150 ms before stimulus onset and until a response is given. In the *drawings* set, participants switch between animacy and size, in the *shapes* set between color and shape, and in the *numbers* set between parity and magnitude. Participants indicate their responses by pressing the A or L key. People typically need longer to respond if the task set in the current trial is different from the preceding trial’s task set (*switch* trials, e.g., an animacy trial that is preceded by a size trial) relative to when the task repeats (*repetition* trials, e.g., an animacy trial that is preceded by another animal trial). To allow for an equal number of switches and trials within blocks, each block contains a *start* trial which will be discarded in the analysis. Therefore, each block in the switching tasks contains 81 trials, and the total number of trials per stimuli set is 324. Within each block, the sequence of trials is randomized, with equal distributions of each stimulus, task set, correct response, and trial type (switch vs. repetition). In addition, the randomization procedure prevents more than four response repetitions in a sequence (excluding the start trial).

#### Dual tasks (high attentional control demands condition)

Participants are asked to classify a stimulus according to two task sets at the same time (e.g., classify drawings based on their animacy and their size). These tasks are presented and performed simultaneously within each trial. Participants respond to one of the two task sets by pressing A or S (animacy, color, and parity), and to the other task set (size, shape, and magnitude) by pressing K or L. Each stimulus is preceded by a blank interval of 150 ms. Within each block, the sequence of trials is randomized, with equal distributions of each stimulus and correct response.

### Training and transfer gains

All cognitive tasks begin with an instruction and blocks of practice trials during which feedback on the accuracy of the responses is displayed during a 250 ms response-stimulus interval. Each of the following test blocks is preceded by a brief instruction to remind participants of the task and stimulus–response bindings, and a visual countdown counting down from 3. No feedback is given during the test blocks. Participants are instructed to take two 3–5 min breaks, one after the first and one after the second third of tasks. If they need to, participants will further be able to take short breaks between tasks as well as between blocks of trials within tasks.

#### Training and near transfer gains

Training gains of the four groups will be compared by using test versions of the simple RT, choice RT, switching, and dual tasks; near transfer will be assessed by measuring gains in these test versions of the training tasks of the respective other groups. These test versions will be identical to the training versions except that no feedback will be provided during the blocks of test trials and that each trial is followed by a 100 ms blank interval. The four blocks of the choice RT task will be split so that the first two blocks will be administered before, and the second two blocks will be administered after the switching task. Each choice RT block is preceded by 5 practice trials, and the first block of each of the simple RT, switching and dual tasks are preceded by 10 practice trials. The test versions of the training tasks present the same trial order for all participants but are pseudo-randomized using the same constraints as the training versions of these tasks. Dependent measures will be accuracy and RT.

#### Far transfer

##### Working memory

*Updating *[36, 43]. Participants are asked to memorize an initial set of single digits presented simultaneously for 3750 ms in three boxes on the screen, followed by a 250 ms blank interval. In the following updating phase, these digits are substituted by new digits and displayed for 1250 ms each, with a 250 ms blank interval between substitutions. Participants are to keep track of the most recent digit in each of the three boxes. Across all test trials, but not within each trial, each box is updated equally often. The same box can be updated multiple times within one trial. After 0, 3, or 6 substitutions, question marks appearing sequentially in each box prompt participants to recall the most recent digit for the respective box by pressing the respective number key. Each trial is followed by a 100 ms blank interval. Participants complete 18 test trials (6 per number of substitutions), split into two blocks of 9 trials each, and preceded by 3 practice trials. Accuracy will serve as dependent variable.

*Binding *[6, 44]. Participants are asked to memorize and later recognize sets of associations between colored triangles and their locations in a 4 × 4 grid. In each trial, 3 to 5 triangles are presented sequentially for 900 ms, followed by a 100 ms blank interval. After memorization, each association is probed using the position as cue, and participants are to indicate whether the association shown matches one of those presented during the memorization phase by pressing the left (“yes”) or right (“no”) arrow key. Half of the probes are matches (i.e., triangles in the correct color in the correct location), and the other half are intrusions (triangles of a color that was associated with a different location). Each color and each location occur equally often across trials, with no repetitions within trials. Probe locations for intrusions are distributed randomly. Each trial is followed by a 100 ms blank interval. Participants complete 24 test trials (12 per set size), split into two blocks of 24 trials each, and preceded by 6 practice trials. As dependent variable, we will compute the discrimination parameter *d*’, which is the difference between *z*-transformed hit rates to match probes and *z*-transformed false alarm rates to intrusion probes.

*Continuous Reproduction *[45]. Participants are asked to memorize the orientations of five isosceles triangles presented simultaneously and spaced equally in a circle on the screen for 1200 ms. Each trial begins with a fixation cross displayed for 500 ms. After a 900 ms retention interval, one probe triangle is presented in a random orientation. Participants are to reproduce the original orientation by moving the mouse and left click to submit the response. Participants complete 100 test trials, split into two blocks of 50 trials each, preceded by 10 practice trials. We will use the signed response error (degrees) to estimate parameters for working memory capacity and/or precision, using the standard mixture model [46] or the swap-model [47]. Model comparisons based on the Aikaike Information Criterion (AIC) and Bayesian Information Criterion (BIC) will determine best model fit and, thereby, the specific indices we will use for this task.

##### Inhibitory control

*Go/No-Go *[48]. Participants are asked to press space if a square appears (“go” trials) and withhold their response if a diamond appears (“no-go” trials). After displaying a fixation cross for 250 ms, stimuli are presented centrally on the screen for up to 2000 ms or until a response is given. Participants complete 216 go trials and 72 no-go trials (288 in total), split into two blocks of 144 trials each and preceded by 12 practice trials. As dependent variable, we will compute the discrimination parameter *d*’, which is the difference between *z*-transformed hit rates to go trials and *z*-transformed false alarm rates to no-go trials.

*Number Stroop *[39, 49]. Participants are asked to indicate how many digits are displayed centrally on the screen by pressing the number keys 1 through 4. The 1 to 4 identical digits displayed can be congruent (e.g., “22” or “4444”) or incongruent with their number (e.g., “3” or “2222”). Each stimulus appears equally often within each condition. Each trial is followed by a 100 ms blank interval. Participants complete 216 congruent trials and 72 incongruent trials (288 in total), split into two blocks of 144 trials each and preceded by 12 practice trials. As dependent variable, we will compute the congruency effect, that is, the difference in log-transformed RTs between incongruent and congruent trials. Only RTs to correct responses will be used, and RT will be trimmed by excluding any RTs more than 3 median absolute deviations away from the overall median (determined per participant and condition).

*Simon *[36, 50]. Participants are asked to indicate the color of a green or red circle presented on the left or right of the screen by pressing A for green circles or L for red circles, respectively. The location of the circle and the location of the response key can be congruent (e.g., a red circle presented on the right) or incongruent (e.g., a red circle presented on the left). Each stimulus appears equally often within each condition. Each trial is preceded by a fixation cross displayed for 250 ms. Participants complete 216 congruent trials and 72 incongruent trials (288 in total), split into two blocks of 144 trials each and preceded by 12 practice trials. The dependent variable is the RT congruency effect computed the same as for the congruency effect in the Stroop task, with the same trimming procedure.

##### Reasoning

*Matrix Reasoning*. We will use the short form of Raven’s Advanced Progressive Matrices [RAPM; 51–53]. Participants are asked to identify the missing element that completes a pattern vertically and horizontally by choosing 1 of 8 alternatives. The task consists of 12 trials with a 15 min time limit, preceded by 2 practice trials. The dependent variable is the proportion of correctly solved problems out of 12.

*Paper Folding *[54]. Participants are presented an illustration of a folded piece of paper with markings indicating where the paper is folded and where a hole is punched through. Participants are asked to select the 1 out of 5 options that correctly shows how the paper looks when completely unfolded. The task consists of two parts, each consisting of 10 trials with a 3 min time limit, preceded by 1 practice trial. The dependent variable is the proportion of correctly solved problems out of 20.

*Letter Sets Part II *[54]. In each trial, participants are presented five sets of four letters, with four of these sets following a common logical pattern. Participants have to identify the deviating letter set. The task consists of 15 trials with a 7 min time limit, preceded by 2 practice trials. The dependent variable is the proportion of correctly solved problems out of 15.

##### Everyday cognitive functioning

To assess transfer to everyday cognitive functioning, we will use two self-report questionnaires and one performance-based test. The *Cognitive Failures Questionnaire* [CFQ; 55–57] is a 25-items self-report measure of failures in perception, memory, and motor function using a 5-point Likert scale. The dependent variable is the summed total score with higher scores indicating more failures (max. 100). The *Questionnaire d’Auto-évaluation de la Mémoire* [QAM short version; 58, 59] is a 11-item self-report measure of difficulties in completing everyday memory tasks on a 6-point scale (from “never” to “always experiencing difficulties”). The dependent variable is the summed total score with higher scores indicating more difficulties (max. 60). As a performance-based measure, we will use an updated and adapted version of the *Everyday Problems Test* [EPT; adapted from 60], in which participants are to solve tasks resembling problems in everyday life such as calculating measurements for recipes. Participants have to choose the correct answer out of 4 alternatives within 1.5 min for each of 14 problems, with the remaining time being visualized with a timer displayed on top of the screen. After each response, participants press a key to proceed to the next item. The dependent variable is the proportion of correctly solved problems (out of 14).

### Individual characteristics

To assess individual characteristics that may potentially affect training and transfer gains, participants will be asked to complete a number of short questionnaires inquiring about their sociodemographic background (e.g., age, gender, education), their previous cognitive training experience through commercial products and research participation, their motivation to participate in the study, computer and internet literacy [based on 61], self-rated physical health [based on 60], general health [62], depression and anxiety [Depression, Anxiety and Stress Scale—21 Items, DASS-21; 63–66], instrumental activities of daily living [Activities of Daily Living-Prevention Instrument, ADL-PI; 67], leisure activities [adapted from 68], active driving [adapted from 69], personality [Big Five Inventory, BFI; 70–72], perseverance [Short Grit Scale, Grit-S; 73, 74], cognition-related beliefs [Theories of Intelligence Scale, TIS; 75; German translation from 38], training-related self-efficacy [modeled after the Exercise Self-Efficacy Scale, EXSE; 76; German translation from 5], and general self-efficacy [GSE; 77–79].

After training, we will also ask participants to self-report their perceived training benefits in the trained tasks, untrained tasks, and everyday life [5, 6], the perceived difficulty of the training tasks, and to review the training intervention (enjoyment and general feedback on participation). In addition, we will measure daily factors potentially affecting cognitive task performance at each visit to the laboratory and at the beginning of each training session. This brief questionnaire will include one question each on sleep quality ["How well did you sleep tonight?", 80, 81] on a 5-point Likert scale (1: “very poorly”, 5: “very well”), stress ["How stressed or rushed do you feel today?" 82] on a 7-point Likert scale (1: “not at all”, 7: “very strongly”), agreement to statements regarding motivation (“I am highly motivated to work on the cognitive tasks today”) and subjective control of attention ["Today, I can concentrate on one activity for a long time if necessary", 83] on a 8-point Likert scale (1: “Does not apply at all”, 8: “Applies very well”), and six questions to assess agreement to statements related to current mood [84–86] on 7-point Likert scales (1/7: “very tired”/ “very awake”; “very content”/ “very discontent”; “very nervous”/ “very calm”; “very alert”/ “very sleepy”; “very bad”/ “very good”; “very relaxed”/ “very tense”).

### Data analysis

#### Missing data and data treatment

We aim for including as many data as possible from participants who meet the eligibility criteria. In general, we intend to exclude participants only when necessary for the given analysis. Because this is a mechanistic study [28] and we intend to use latent-variable modeling, we plan to conduct per-protocol analysis. Therefore, for analyses beyond pretest data, we intend to include only participants who have completed at least one posttest session and 70% of training sessions. We will not impute any data.

We will apply transformations to this data as needed (e.g., *z*-transformations) based on the properties of the collected data (e.g., non-normal distribution). Such transformations and the reason for performing the transformations will be documented and reported. We intend to operationalize task performance in the training tasks using the drift rate. If using the drift rate is not possible because the data properties prevent us from fitting diffusion models, we will use mean RTs, switching and dual tasking costs. In addition, we will analyze training effects on the other parameters of the drift diffusion model, in particular boundary separation and non-decision time, and test how changes in these parameters relate to changes in the transfer tasks.

#### Computational and statistical modeling

We will estimate the diffusion model parameters for performance in the training tasks. Simulation studies have shown that the EZ/Robust EZ diffusion model [87, 88] is adequate for a wide range of applications [89]. Indeed, the Robust EZ model [88] was suitable for a preliminary analysis of choice RT data from a previous study [39]. However, depending on the properties of the acquired data set, we may need to use other diffusion model implementations with different estimation procedures such as the hierarchical diffusion model [90]. The parameters of the diffusion model that are directly relevant to our main hypotheses are drift rate, response caution, and non-decision time.

We plan to use latent change-score models to evaluate training and transfer gains, modeled after those previously reported by Schmiedek et al. [91]. To compare latent change between the different conditions, we will run these models for multiple groups simultaneously. We will first test all four levels of measurement invariance (configural, metric, scalar, and strict invariance) across study sites, times, and groups. Next, we will test whether latent change differs between two conditions by comparing model fit when this parameter is allowed to vary freely or is fixed to be the same between groups.

If the models fail to converge, we will use Bayesian linear-mixed effects modeling to account for variance from multiple individuals and tasks following the same procedures as in our previous work [5, 6]. More specifically, if using linear mixed-effects models, we would include fixed effects of group (4 training conditions) and age (continuous variable from 18 to 85 years), and random effects of participant (individual ID) and task (e.g., drawings, numbers, shapes for the training tasks).

#### Inference criteria

We will follow conventional guidelines for interpreting Bayes factors [92] and regard BFs between 0.33 and 3 as reflecting ambiguous evidence. Model fit of latent-variable models will be evaluated using the root mean square error of approximation (RMSEA) alongside its 90% confidence interval, the standardized root-mean-square residual (SRMR), and comparative fit index (CFI). We will follow conventional standards indicating good fit [93]: RMSEA < 0.06, SRMR < 0.08, and CFI > 0.95. In addition, the chi-square statistic (χ^2^) will be reported. Differences in model fit will be examined using chi-square difference tests (Δχ^2^) and by examining the differences in AIC and BIC.

#### Hypothesis 1 (training gains)

To investigate gains in the trained domains, we will model latent change from (1) pretest to posttest, (2) pretest to follow-up, and (3) posttest to follow-up. Specifically, we will use performance in the three training tasks (drawings, numbers, shapes) as manifest variables loading on a latent variable each at pretest and posttest (or follow-up). To assess whether training gains in the experimental conditions exceed mere test–retest effects, we will run these latent-difference score models for multiple groups simultaneously (e.g., comparing dual task training to active control training) and test whether latent change differs between each experimental condition and the active control condition. We will compare each experimental condition separately to the active control condition. For all three experimental conditions, we expect positive latent change scores significantly larger than those observed in the active control condition, indicating improvement from (1) pretest to posttest and (2) pretest to follow-up. If training effects are maintained after training, there should be no negative latent change from posttest to follow-up.

#### Hypotheses 2 and 3 (transfer gains)

To test for group differences in training-induced gains in the untrained near and far domains, we will run latent-difference score models for the transfer domains (e.g., using the three working memory measures to load on a latent working memory factor at each pretest and posttest, or follow-up) and test whether latent change differs between each experimental condition and the active control condition. Positive latent change in the experimental conditions significantly larger than that in the control condition indicates transfer gains. The absence of negative latent change from posttest to follow-up will indicate that transfer gains are maintained after training.

#### Hypothesis 4 (attentional control demands)

To test whether increasing attentional control demands lead to larger transfer effects, we will run the same models as above but compare each experimental condition to each other. We expect training groups with higher attentional control demands to show significantly higher latent change scores than those groups with lower attentional control demands (i.e., dual task > switching task > choice RT task).

#### Hypothesis 5 (underlying mechanism)

To test whether training-induced changes in drift rate predict near and far transfer, we will combine the latent-change models for training and transfer domains and use the latent change in the training domain as predictor of the latent change in the transfer domain. We will compare (1) each experimental condition separately to the active control condition, and (2) the experimental conditions to each other. We expect that training-induced changes in drift rate are most strongly related to the size of transfer effect relative to the active control conditions if acquired in a training context with relatively higher attentional control demands (i.e., dual task > switching task > choice RT task). If the multigroup models fail to converge, we will attempt modeling the relationships for each group separately and for all groups together.

## Discussion

With this study, we aim to identify the cognitive processes changed by training speed tasks with varying attentional control demands across the adult lifespan. The results of this study will yield theoretical and practical implications. At the theoretical level, knowledge about the mechanisms underlying the success of processing speed training interventions will substantially advance understanding of individual differences and plasticity of cognitive abilities. By shifting the focus from the heated yet stagnating debate of whether cognitive training “works” [94] toward the more fruitful question of why some interventions are more successful than others [3], this study will enable a more productive debate. The use of diffusion modeling, a computational approach, will lead to developing a more precise theory of the cognitive processes involved in and affected by processing speed training. Critically, this project will move the cognitive training literature forward by testing a mechanism of transfer that so far has been neglected: the improved rate of evidence accumulation.

At the practical level, this project answers the WHO’s call for the development of evidence-based interventions that are accessible to everyone [95]. Cognitive training interventions are easy to administer, highly affordable, and can be distributed quickly. However, the current lack of knowledge about how the more successful interventions work, and why others do not, yields the risk of opportunity costs outweighing the potential benefits. We hope that our findings will provide a pathway to the development of powerful and, importantly, reliably successful cognitive training interventions. By identifying the mechanisms of training success across the adult lifespan, the results of this study can critically inform how interventions can be tailored to be maximally effective in counteracting age-related declines for people of all ages.

There are also a few potential limitations of this study. As this is an elaborate study that requires participants to invest a substantial amount of their time, we anticipate some attrition during the study. The sample size we aim for allows for some attrition (~15 to 20%), but we cannot predict how many participants will ultimately drop out. Higher than expected study drop-out could thus become a shortcoming of our study. Furthermore, due to this study being conducted across multiple sites in three different languages, findings may diverge between the sites, possibly due to differences in drop-out rate, cultural differences, language differences or other unforeseeable differences between the three sites. By adhering to the common experimental protocols, using carefully translated task instructions and study materials checked by native speakers, and regular meetings between the research teams of the three sites, we aim to minimize any of these potential site differences. Yet, on balance, the multi-site character of this study is of great added value and will allow for further-reaching interpretations of the results, which is worth taking the risk of possible site differences.

Taken together, the results of this study can contribute to identifying the cognitive processes that underpin training-induced cognitive change across the adult lifespan. This study goes substantially beyond previous research by using diffusion modeling to estimate theoretically and psychologically meaningful information processing components involved in performing the training tasks. As yet, no study has tested which diffusion model parameters underlie the benefits of processing speed training across adulthood. Combining diffusion and latent-change modeling to predict transfer and investigate the impact of attentional control demands during training is a novel and innovative approach that may explain why selective training interventions appear to be successful, whereas most other approaches failed to yield meaningful cognitive benefits. A better understanding of how processing speed training affects these cognitive mechanisms across the adult lifespan will enable researchers to maximize the effectiveness of cognitive training during adulthood in producing broad transfer to psychologically meaningful everyday life outcomes.

## Data Availability

Experimental tasks and non-proprietary stimulus materials will be shared on www.tatool-web.com and the Open Science Framework (https://osf.io/umzes). The study team will have exclusive use of the data until the main research findings are published. Following publication of the data, the anonymized data that can be ethically shared will be shared on the Open Science Framework (https://osf.io/umzes). Demographic data that will potentially allow for re-identifying individuals will be stored separately with controlled access. For these data, the Principal Investigators (C.C.v.B., T.S., and S.B.) will jointly review applications to access the data and make the decision on whether to supply research data to potential applicants. Data will then be released on a case-by-case basis.

## Abbreviations

RT: Reaction time
CFQ: Cognitive Failures Questionnaire
QAM: Questionnaire d’Auto-évaluation de la Mémoire
EPT: Everyday Problems Test
DASS-21: Depression, Anxiety and Stress Scale-21 Items
ADL-PI: Activities of daily living-prevention instrument
BFI: Big five inventory
Grit-S: Short Grit Scale
TIS: Theories of Intelligence Scale
EXSE: Exercise Self-Efficacy Scale
GSE: General Self-Efficacy Scale
MoCA: Montreal cognitive assessment
CRIUGM: Centre de Recherche de l’Institut Universitaire de Gériatrie de Montréal
RAPM: Raven’s advanced progressive matrices
AIC: Aikaike information criterion
BIC: Bayesian information criterion
RMSEA: Root mean square error of approximation
SRMR: Standardized root-mean-square residual
CFI: Comparative fit index

## Footnotes

1 Participants excluded during pretest based on their MoCA score will be reimbursed for participation in the pretest.
